# An *In Silico* Approach for Evaluating a Fraction-Based, Risk Assessment Method for Total Petroleum Hydrocarbon Mixtures

**DOI:** 10.1155/2012/410143

**Published:** 2012-02-08

**Authors:** Nina Ching Y. Wang, Glenn E. Rice, Linda K. Teuschler, Joan Colman, Raymond S. H. Yang

**Affiliations:** ^1^National Center for Environmental Assessment, Office of Research and Development, U.S. Environmental Protection Agency, Cincinnati, OH 45268, USA; ^2^Chemical, Biological and Environmental Center, SRC, Inc., Syracuse, NY 13212, USA; ^3^Quantitative and Computational Toxicology Group, Department of Environmental and Radiological Health Sciences, College of Veterinary Medicine & Biomedical Sciences, Colorado State University, Fort Collins, CO 80523, USA

## Abstract

Both the Massachusetts Department of Environmental Protection (MADEP) and the Total Petroleum Hydrocarbon Criteria Working Group (TPHCWG) developed fraction-based approaches for assessing human health risks posed by total petroleum hydrocarbon (TPH) mixtures in the environment. Both organizations defined TPH fractions based on their expected environmental fate and by analytical chemical methods. They derived toxicity values for selected compounds within each fraction and used these as surrogates to assess hazard or risk of exposure to the whole fractions. Membership in a TPH fraction is generally defined by the number of carbon atoms in a compound and by a compound's equivalent carbon (EC) number index, which can predict its environmental fate. Here, we systematically and objectively re-evaluate the assignment of TPH to specific fractions using comparative molecular field analysis and hierarchical clustering. The approach is transparent and reproducible, reducing inherent reliance on judgment when toxicity information is limited. Our evaluation of membership in these fractions is highly consistent (˜80% on average across various fractions) with the empirical approach of MADEP and TPHCWG. Furthermore, the results support the general methodology of mixture risk assessment to assess both cancer and noncancer risk values after the application of fractionation.

## 1. Introduction

Contamination of the environment by petroleum products including crude oil, lubricating oils, and a wide variety of fuels is widespread. Typically, these petroleum products are complex mixtures containing hundreds to thousands of different hydrocarbon compounds, including aliphatic compounds (e.g., straight-chain, branched-chain, and cyclic alkanes and alkenes) as well as aromatic compounds (e.g., benzene and alkyl benzenes, polycyclic aromatic hydrocarbons). Once released into the environment, the composition of a petroleum hydrocarbon mixture can change due to weathering (http://facstaff.gpc.edu/~pgore/geology/geo101/weather.htm). During chemical weathering, components of petroleum mixtures can degrade and partition, such that the more soluble or volatile compounds can be readily transported to other environmental media and locations, while the relatively nonmobile and recalcitrant components (i.e., the weathered products) remain near the point of release. Thus, the actual petroleum hydrocarbon mixtures to which a population might be exposed will vary with the petroleum product released, environmental conditions, elapsed time following release, and exposure medium.

Assessment of human health risks associated with petroleum-hydrocarbon-contaminated sites routinely begins with an analysis of “total petroleum hydrocarbons” (TPHs). TPHs are loosely defined hydrocarbon mixtures. While the components included in a mixture of TPHs depend on the method of analysis and chemical nature of the TPH contaminating material (e.g., jet fuels, gasoline, etc.), TPHs typically represent the total mass of hydrocarbons without identifying specific individual compounds. As TPHs is not a consistent entity, the assessment of health effects and development of toxicity criteria such as reference doses (RfDs) and cancer slope factors for such complex mixtures as a whole are problematic (the RfD is an estimate of the dose of daily exposure to a substance (with uncertainty spanning perhaps an order of magnitude) for a human population (including sensitive subgroups) that is likely to be without an appreciable risk of deleterious effects during a lifetime [1]).

**Figure 1:**
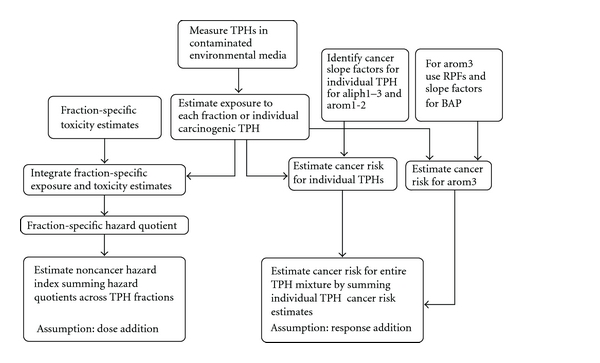
Approach to estimate TPH cancer risks and noncancer Hazard Indices using component and mixture fraction methods. Whole mixture toxicity data are not available for the many, highly variable TPH mixtures of concern that are found at different sites, and as a result, both MADEP and TPHWG have proposed a fractional and subsequent surrogate/component-based approach for the risk assessment of TPHs. This figure shows that following the measurement of TPHs in either soil or water, both MADEP and TPHWG recommend that exposure estimates be developed for each fraction. Relying on surrogates for each fraction, Figure 1 shows that whole mixture risk assessments can be done using data directly on the mixture of concern, on a sufficiently similar mixture, on a mixture's fractions, or on a subset of its components.

As previously mentioned, both the Massachusetts Department of Environmental Protection (MADEP) and the Total Petroleum Hydrocarbons Criteria Working Group (TPHsCWG) recommended a “fraction-based approach” for assessing human health risks associated with TPH exposures [2–8]. Both MADEP and TPHCWG divide the aliphatic TPHs into three aliphatic fractions designated as aliph1–3 (Table 1). MADEP grouped the entire range of aromatics from C9–C32 into a single fraction for the assessment of oral noncancer toxicity and divided the fraction into C9–C18 and C19–C32 fractions for the assessment of inhalation noncancer toxicity. In contrast, TPHCWG divided the aromatic compounds into three individual aromatic fractions designated as arom1–3. Following measurements to determine the concentrations of the six fractions present at a given site, human exposure to the individual fractions can be estimated (Figure 1).

 The noncancer health risk associated with each fraction is then predicted based on the dose-response function of a fraction-specific surrogate chemical(s). The underlying assumption is that each member of a fraction is toxicologically similar and shares a common mode of action (MOA). USA EPA [9] defines “similar components” as single chemicals that cause the same biologic activity or are expected to cause the same type of biologic activity based on chemical structure. These components also may be expected to have comparable kinetic characteristics and toxicity. A Hazard Index based on an assumption of dose addition is then calculated to assess potential noncancer health risks using both chronic and subchronic surrogate toxicity values [9].

The cancer health risks associated with each fraction can be estimated assuming either response addition for all fractions but arom3 or dose addition for a subset of TPHs in the arom3 fraction. For individual TPHs that have cancer slope factors, the products of the TPH exposures and the cancer slope factors are summed to estimate cancer risks posed by those compounds under an assumption of response addition. For groups of carcinogenic TPHs assumed to act through a common toxic mode of action, the relative potency factor approach is used. This approach requires both the existence of toxicological dose-response data for at least one component of the mixture, (i.e., the index chemical), and scientific judgment that the toxicity of the other individual compounds in the mixture are toxicologically similar [9]. Using this approach, some polycyclic aromatic hydrocarbons assigned to the arom3 fraction are assumed to be dose-additive. These hydrocarbons have been assigned a relative potency factor. The relative potency factors for each compound are combined with the compound-specific-intake estimates to calculate an index chemical equivalent dose. These equivalent doses are summed, and the total equivalent dose is compared to a cancer slope factor for the index chemical (i.e., benzo[a]pyrene); cancer risk for the fraction is estimated based on this comparison [10].

As discussed by Teuschler [11], this approach to risk assessment of TPHs “illustrates a flexible method for characterizing TPH exposures that reflects differences in chemical composition across various sites and provides a reasonable method for calculating potential health risks.” We summarized the conceptual basis of the MADEP/TPHCWG approach in Figure 1 principally based on the natural and logical course of chemical extraction, separation, and analysis. Such chemical analyses are intimately associated with the physicochemical properties of the component chemicals present in the TPH mixture (see http://hhpprtv.ornl.gov/issue_papers/ComplexMixturesofAliphaticandAromaticHydrocarbons.pdf for full analysis).

For this chemical mixtures risk assessment approach, the accurate assignment of the individual TPHs into specific fractions is critical. Fraction or membership assignments have been based on physicochemical properties (including molecular structure such as aromatic versus aliphatic), analytical data, prevalence in the environment (e.g., fate and transport), and toxicological properties. The analytical data are based on the number of carbon atoms (C) in the compounds comprising the fraction or equivalent carbon (EC) number index. Inaccurate assignments of TPHs into fractions could lead to erroneous evaluations of the overall noncancer and cancer risks. Table 1 summarizes noncancer toxicity values for various TPH fractions derived by MADEP.

Because *in silico* molecular modeling or computational modeling is often employed for analysis, interpretation, and visualization of heterogeneous datasets from various sources (e.g., fractions) [12–15], we used an *in silico* approach in this paper to systematically examine the assignment of 111 selected TPH components into specific fractions. These examinations are based on a molecular modeling approach achieved through the application of comparative molecular field analysis (CoMFA^2^), which is based on three-dimensional (3D) shape, electrostatic and hydrogen bonding characteristics (assuming similar MOAs and molecular targets) and then evaluated by hierarchical clustering. CoMFA was specifically selected for capturing molecular interactions between TPHs and their potential common molecular targets. TPHs with common molecular targets will likely be clustered together based on their CoMFA descriptors, and these clustered TPHs are expected to be assigned to a specific fraction (e.g., arom3 TPHs bind to aryl hydrocarbon receptor). Additional analysis by structure-activity relationship can also be utilized to evaluate membership consistency within a fraction. This approach was applied to examine the underlying association of chemicals within each fraction, thereby providing information relevant to the assumed common toxicity of individual TPHs in each fraction. We compared the assignments predicted using this method to those developed by MADEP.

## 2. Materials and Methods

### 2.1. Selection of Individual TPHs

**Figure 2:**
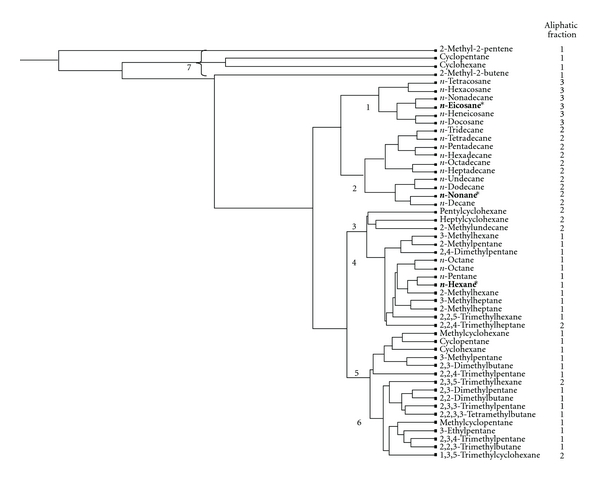
Hierarchical cluster analysis based on CoMFA for all aliphatic fractions. Surrogate chemicals proposed by MADEP [3] are shown in bold and with asterisks.

**Figure 3:**
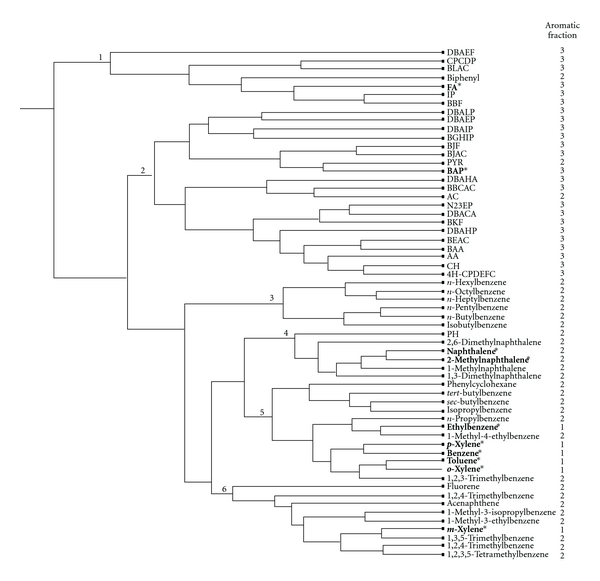
Hierarchical cluster analysis based on CoMFA for all aromatic fractions. Proposed surrogate or component chemicals are shown in bold and with asterisks. DBalP: Dibenzo[a,l]pyrene, BlAC: Benzo[l]aceanthrylene, IP: Indeno[1,2,3-c,d]pyrene, FA: Fluoranthene, CPcdP: Clclopenta[c,d]pyrene, DBacA: Dibenzo[a,c]anthracene, BbcAC: 11H-Benzo[b,c]aceanthrylene, AA: Anthanthrene, DBahA: Dibenzo[a,h]anthracene, BjAC: Benzo[j]aceanthrylene, BeAC: Benzo[e]aceanthrylene, BjF: Benzo[j]fluoranthene, BaA: Benzo[a]anthracene, N23eP: Naphtho[2,3-e]pyrene, BghiP: Benzo[g,h,i]perylene, Pyr: Pyrene, PH: Phenanthrene, AC: Anthracene, DBaiP: Dibenzo[a,i]pyrene, DBahP: Dibenzo[a,h]pyrene, DBaeF: Dibenzo[a,e]fluoranthene, DBaeP: Dibenzo[a,e]pyrene, BkF: Benzo[k]fluoranthene, BbF: Benzo[b]fluoranthene, BaP: Benzo[a]pyrene, CH: Chrysene, 4H-CPdefC: 4H-Cyclopenta[d,e,f]chrysene.

The selection of aliphatic and aromatic TPHs was based on their abundance in contaminated sites (e.g., composition and type of fuels) and/or known qualitative significant toxicity (e.g., endpoints or critical effects). Overall, 51 aliphatics and 60 aromatics were chosen based on the available toxicity information as the two main datasets for subsequent analysis and validation. All selected aliphatic and aromatic TPHs are listed in Figures 2 and 3, respectively. 

### 2.2. Carbon Atoms (C) and Equivalent Carbon (EC)

The EC number is measured by comparing a compound's retention time in gas chromatography to that of various *n*-alkanes [3, 8]. This index is equivalent to the retention time of the compounds on a boiling-point gas chromatography (GC) column (nonpolar capillary column) normalized to the *n*-alkanes. For example, benzene, a C6 aromatic compound, has an EC of 6.5 because its boiling-point and GC retention time are approximately halfway between those of *n*-hexane (C6, EC6) and *n*-heptane (C7, EC7). Physical and chemical properties of hydrocarbons that are useful in predicting fate and transport including vapor pressure, solubility, partition coefficient, and Henry's Law constants are predictably related to the EC and can be estimated using algorithms. Both MADEP and TPHCWG have adopted this method for the fractionation of TPHs. 

### 2.3. In Silico Approach

Following the selection of the TPHs, the analysis was conducted in three steps as follows: (1) molecular modeling; (2) CoMFA analysis; (3) hierarchical clustering analysis. Each step illustrates the characteristics and physicochemical properties of hydrocarbons within each fraction.

**Step 1**

*Molecular Modeling. *Initial structures of the 111 TPHs were built using the Sketch Molecule module in Sybyl 8.0 (Tripos, Inc., St. Louis, MO, USA), and energy was subsequently minimized to yield a stable conformation using the MMFF94 force field and electrostatic charges [16]. The 51 aliphatics and 60 aromatics were processed and used as two separate datasets for further analyses. Each dataset was aligned using its common core structure (i.e., pentane for aliphatics; and benzene for aromatics) prior to COMFA analysis.

**Step 2**

*Comparative Molecular Field Analysis (CoMFA)*. Because most TPHs are postulated to have similar toxic effects or modes of action (e.g., toxicity, binding affinity, etc.), a CoMFA [17] was conducted based on the current understanding of mixtures risk assessment [9, 10]. CoMFA is a commonly used 3D quantitative structure-activity relationship technique from which inferences can be made about chemicals of interest based on data from known active molecules. In general, to apply CoMFA to a group of chemicals, all that is required for the analysis is the biological activity (e.g., IC_50_) and the 3D structures of the molecules assuming common molecular target(s). However, for this paper, only the CoMFA descriptors were needed for further hierarchical clustering; no biological activity data were used. It was not our intent to develop quantitive structure-activity relationship models. The 3D structures of the molecules were constructed as described in *Step 1.* Briefly, TPHs were placed in a 3D grid with 2-Å spacing encompassing all of the chemicals. At each grid point, both steric energy and electrostatic energy were measured for each chemical by a probe atom (sp^3^-hybridized carbon with +1 charge). All steric and electrostatic energies were set to the default cutoff value of 30 kcal/mol. All other parameters for CoMFA were set to the default values in Sybyl 8.0. CoMFA values for each compound were computed according to procedures in the software manual (Sybyl 8.0, Tripos, Inc.).

**Step 3**

*Hierarchical Clustering Analysis. *Once the CoMFA values of all selected TPHs were calculated, two heuristic hierarchical clustering analyses were separately conducted for aliphatic and aromatic groups (Figures 2 and 3). In general, a hierarchical clustering analysis attempts to find groupings within a set of data. Based on the Euclidean distance between points, a dendrogram showing the similarity/dissimilarity of clusters at increasing levels of detail is displayed (Sybyl 8.0, Tripos, Inc.). A hierarchical clustering analysis is performed based on the CoMFA results from Step 2 above. Initially, each dot (i.e., chemical) on the rightmost column of the dataset of TPHs can be considered as a basic cluster. The next nearest pair of clusters is merged (indicating similarity in chemical structure and potential biological interactions), then the next nearest (to the left), and so forth until there is only one cluster containing all the dots (chemicals) and branches. The overall hierarchical clustering process is captured as a dendrogram or inverted tree (note: for this paper, Figures 2 and 3 have been rotated 90°counterclockwise to facilitate viewing). The rotated dendrogram should be read from the right to the left, where each node at the rightmost end represents a chemical, and the central branch at the next merging point represents the entire dataset for one cluster. The lengths of the horizontal lines in the dendrogram provide relative qualitative information about the linkage distance (e.g., similarity) between various clusters. For instance, clusters represented by long unbranched strands are strongly separated from other clusters (Figures 2 and 3).

The main property used to determine the distances on which clustering operates is CoMFA descriptors. Once the CoMFA column (an array of 3D descriptors) is generated, the linkage method for hierarchical clustering is *Complete* as recommended by the software manual (Sybyl 8.0, Tripos, Inc.). In general, the *Complete* linkage yields the fewest singletons and the most balanced distribution of points among clusters (Sybyl 8.0, Tripos, Inc.). Because clusters based on the *Complete* linkage method are comprised of only similar components, this represents a significant advantage over the other three linkage methods. Thus, we presented results from the *Complete* linkage method only (other linkage methods were attempted but no major difference in overall pattern of clustering was found; data not shown). The full details of algorithms can be found in the software manual (Sybyl 8.0, Tripos, Inc.).

## 3. Results and Discussion

The dendrogram results are displayed in Figures 2 and 3 of aliphatics and aromatics, respectively. The fraction assignment developed by MADEP is in the first column of Table 1 and is also indicated in the rightmost column in Figures 2 and 3. The clusters are labeled by numbers in Figures 2 and 3. Based on intrinsic chemical structures (e.g., cyclic versus single chain, and branched versus unbranched hydrocarbons) and the final hierarchical clustering, seven clusters were manually assigned for the aliphatic fractions (aliph1–3; numbers 1–7 in Figure 2) based on the separation of clusters and degree of branching, and six clusters were manually assigned for the aromatic fractions (arom1–3; numbers 1–6 in Figure 3). Consistency of the fraction assignments for the TPHs was assessed based on the assignment of the global clusters.

### 3.1. Clustering Results of Aliphatic TPHs

For the aliphatic group containing 51 component chemicals, it is apparent that alkenes (Cluster number 7) are strongly separate and are most dissimilar to the rest of TPHs that are mostly straight and branched alkanes (Figure 2). If considering all alkenes as a global cluster on its own, the rest of dendrogram can be grouped into six clusters based on the comparable length of horizontal lines (i.e., CoMFA similarity) as labeled in Figure 2. Based on this classification or grouping, we compared membership of each TPH component within each cluster to its actual fraction assignment by MADEP as indicated in the aliphatic fraction column. In general, Cluster number 1 represents aliph3, Cluster number 2 represents aliph2, and the remaining clusters represent aliph1. Consistency of fraction assignment is summarized in Table 2. The overall consistency with the MADEP fraction assignment is the highest (100%) for aliph3, followed by aliph1 (86.2%) and aliph2 (62.5%). The overall consistency for the three aliphatic fractions is 80.4%. The aliphatic dendrogram suggests that some TPHs assigned by MADEP to aliph2 may be more similar to compounds in aliph1 based on their 2D and 3D physicochemical properties. To determine whether this hypothesis is valid, further hierarchical analysis based on both toxicity values and CoMFA will be necessary. This hierarchical clustering is heuristic in the sense that it depends on physiochemical properties (CoMFA) only. Future work may include a 2-way hierarchical clustering using both CoMFA and a common toxicity endpoint and data which could facilitate direct comparison of underlying toxicity and further evaluation of the proposed surrogate chemical within each fraction.

### 3.2. Clustering Results of Aromatic TPHs

For the aromatic group containing 60 component chemicals, six clusters can be identified based on the leftmost level (Figure 3). Clusters numbers 3–6 can be considered as a global cluster based on the comparable length of horizontal lines (Figure 3). This global cluster consists of arom1 and arom2 TPHs only. Cluster number 1 is the furthest separated from the rest of the clusters, but it is closer to Cluster number 2. Clusters numbers 1 and 2 can be considered as another global cluster, which consists of all arom3 TPHs.

The percent consistency is the highest for arom3 (100%), followed by arom1 (83.3%), and arom2 (66.7%). The overall consistency for the three aromatic fractions is 88.3%. The low consistency percentage within the arom2 fraction can be explained by two noticeable discrepancies for some TPHs as follows. (1) Three TPHs (pyrene, anthracene, and biphenyl) were shown to be strongly clustered with the arom3 fraction Clusters numbers 1 and 2 instead of arom2 Clusters numbers 3, 4, and 6 (Figure 3), and (2) seven TPHs with lower EC indices within the arom2 fraction clustered closely with the arom1 TPHs (e.g., *n*-propylbenzene). Based on the clustering results, we would recommend further analyses for pyrene, anthracene, and biphenyl for this mixtures risk assessment approach, as they may be considered as part of arom3 rather than arom2. As for the low EC TPHs within the arom2 fraction (e.g., *n*-propylbenzne, isopropylbenzene, etc.), there is some suggestive evidence that the cutoff for the arom2 may be changed to EC11 instead of EC9 by including straight and branched propylbenzene and butylbenzene in certain circumstances. This is based on available oral toxicity values for ethylbenzene (EC8.5) and isopropylbenzene (EC9.13), as both have similar lowest-observed-adverse-effect levels (LOAELs) of 291 and 331 mg/kg-day, no-observed-adverse-effect levels (NOAELs) of 97.1 and 110 mg/kg-day, respectively, as well as identical RfDs of 0.1 mg/kg-day. Further details can be found in the U.S. Environmental Protection Agency's Integrated Risk Information System database [1]. A recent two-generation reproductive study with repeated exposure to *n*-butylbenzene suggests that a LOAEL may be identified at 300 mg/kg-day based on hepatotoxicity (e.g., increase liver weight and associated hepatocellular hypertrophy; [18]), which is close to the LOAEL for ethylbenzene based on similar liver toxicity endpoints. Additional toxicity testing and/or risk assessment for the low EC TPHs will be needed to support the deviation from the criteria for the arom2 fraction for these specific TPHs.

As for components that are not typically considered as surrogate chemicals for a specific fraction, components with different and more potent toxicities such as naphthalene and other substituted naphthalenes in arom2 fraction should be assessed separately based on the recommendation of MADEP. We found that all naphthalenes clustered closely (Cluster number 4 in Figure 3), and our results supports the MADEP recommendation. The only inconsistently classified arom1 TPH was *m*-xylene, as it is shown to cluster closely with branched alkylbenzenes (Cluster number 6 in Figure 3). It is uncertain why *m*-xylene did not cluster with either *p* or *o*-xylene. We tentatively hypothesize that there may be different metabolizing enzymes that are position-specific, because the overall shape and volume of *m*-xylene are significantly different from that of *p*- and *o*-xylenes.

### 3.3. Comparison of Fraction Assignments

In general, the assignment of membership in a fraction using our integrated CoMFA/hierarchical clustering approach was consistent with the MADEP assignments based on analytical chemistry for environmental fate and transport. We found that the rate of consistency with the MADEP assignments is >80% on average for the three fractions in the aliphatic and aromatic groups. We believe that CoMFA/hierarchical clustering approach for assigning components or individual TPHs to specific fractions is complementary to the established fraction-based approach for TPH mixture assessment.

## 4. Conclusions

The *in silico* molecular modeling approach presented in this paper is an important contribution to the assessment of risks posed by TPH mixtures and represents the first known case study that applies computational tools to augment a mixtures risk assessment approach. This integrated CoMFA/hierarchical clustering approach allows systematic and objective evaluation of TPH fractions and fractional membership through a repeatable process. The approach is capable of clustering or grouping members within a fraction and assigning membership in a fraction (i.e., a local cluster). This approach can also identify TPHs that belong in other fractions (i.e., global clusters). Finally, the approach is transparent and reproducible, reducing inherent uncertainty in judgments when chemistry and toxicity information is limited or not available.

We used the approach to independently evaluate membership assignments in the TPH fractions that were developed by MADEP. In general, we found the composition of the MADEP fractions to be consistent with results from the CoMFA/hierarchical clustering approach. Concordance between these approaches reduces the uncertainty associated with applications of this mixtures method. However, we also observed some discrepancies between our results and those of MADEP. For instance, MADEP includes the C5–C8 TPHs in the arom1 fraction, but our analysis suggests that some C9-C10 TPHs (e.g., *n*-propylbenzene, isopropylbenzene, and *n*-butylbenzene) in MADEP's arom2 fraction have physicochemical and toxicological properties similar to TPHs in the arom1 fraction, and may therefore be more appropriately assigned to arom1. The application of our approach suggests that additional toxicological evaluations of some “borderline” TPHs may provide useful insight into their assignment to a specific fraction.

Overall, our approach has some limitations in distinguishing one fraction from another (e.g., arom1 from arom2) and in defining a cutoff in terms of carbon or EC number. It is also highly dependent on the available toxicity information, because the clustering results could change with consideration of compound-specific toxicity values. We believe that in the future, development of a two-way hierarchical clustering (i.e., CoMFA and toxicity information) would perform better than the CoMFA-based approach alone using a subset of TPHs within a fraction. Further studies could include hierarchical clustering using both CoMFA descriptors and repeated dose toxicity information (e.g., NOAELs, LOAELs, reference values, and cancer slope factors) as an extra level of filtering for the hierarchical clustering analysis. A refined clustering using both CoMFA and a common toxicity endpoint would facilitate a direct comparison of underlying toxicity and potentially validate the proposed surrogate chemical within each fraction. By taking the information and knowledge gained from both the toxicology and chemistry fields, an empirical approach is available to define similarity and ultimately the grouping for a specified fraction for future risk assessments of TPH mixtures.

 A significant future application of this approach involves assigning toxicologically unknown TPHs to fractions prior to analytical chemistry measurements. Without the actual measurement of EC number or an expert's judgment, one cannot assign a TPH to a specific fraction with confidence. Using this *in silico * approach, toxicologically unknown TPHs can be considered with the rest of the toxicologically known TPHs in all fractions based on CoMFA descriptors, and the cluster/fraction which it most closely associates with can be subsequently identified (i.e., a range of toxicity can be inferred). Based on clustering, one may assign membership in an appropriate fraction based on proximity to the surrogate chemical or the overall clustering/grouping of TPHs in a fraction (i.e., similar components or chemicals within a fraction tend to cluster together). In addition, toxicity of the surrogate chemical within the assigned fraction could serve as the surrogate toxicity for the unknown TPHs. While this transparent and empirical approach can address uncertainty for toxicologically unknown TPHs in a mixture, it cannot predict an actual toxicity value for the TPHs with unknown toxicity. 

## Figures and Tables

**Table 1:** MADEP toxicity values for TPH fractions [3].

Hydrocarbon fraction	Oral (mg/kg/day)	Inhalation (mg/m^3^)
*Aliphatic*		
No.1: C5–C8	0.04	0.2
No.2: C9–C18	0.1	0.2
No.3: C19–C32	2.0	NV^a^

*Aromatic*		
No.1: C6–C8	SC^b^	SC^b^
No.2: C9–C18	0.03^c^	0.05
No.3: C19–C32	0.03^c^	NV^a^

^a^NV: not volatile.^b^SC: used single chemical values (fraction includes benzene, toluene, xylene, and ethyl benzene).  ^c^MADEP grouped the entire range of aromatics from C9–C32 into a single fraction for oral noncancer toxicity.

**Table 2:** Consistency of fraction assignment.

Hydrocarbon fraction	Consistency	Percent consistency
*Aliphatic*		
No.1: C5–C8	25/29	86.2%
No.2: C9–C18	10/16	62.5%
No.3: C19–C32	16/16	100%

Total	41/51	80.4%

*Aromatic*		
No.1: C6–C8	5/6	83.3%
No.2: C9–C18	24/30	66.7%
No.3: C19–C32	24/24	100%

Total	53/60	88.3%

## Abbreviations

C: Carbon
CoMFA: Comparative molecular field analysis
EC: Equivalent carbon
GC: Gas chromatography
NOAEL: No-observed-adverse-effect level
LOAEL: Lowest-observed-adverse-effect level
MADEP: Massachusetts Department of Environmental Protection
MOA: Mode of action
RfD: Reference dose
TPH: Total petroleum hydrocarbon
TPHCWG: Total petroleum hydrocarbon criteria working group.
